# Patient involvement in developing a patient‐targeted feedback intervention after depression screening in primary care within the randomized controlled trial GET.FEEDBACK.GP

**DOI:** 10.1111/hex.13039

**Published:** 2020-04-14

**Authors:** Tharanya Seeralan, Martin Härter, Cornelia Koschnitzke, Michael Scholl, Sebastian Kohlmann, Marco Lehmann, Marion Eisele, Lea‐Elena Braunschneider, Gabriella Marx, Martin Scherer, Bernd Löwe, Julia Luise Magaard, Anna Levke Brütt

**Affiliations:** ^1^ Department of Medical Psychology Center for Psychosocial Medicine University Medical Center Hamburg‐Eppendorf Hamburg Germany; ^2^ Member of the Participatory Research Team Hamburg Germany; ^3^ Department of Psychosomatic Medicine and Psychotherapy Center for Internal Medicine University Medical Center Hamburg‐Eppendorf Hamburg Germany; ^4^ Department of Primary Medical Care Center for Psychosocial Medicine University Medical Center Hamburg‐Eppendorf Hamburg Germany; ^5^ Department for Health Services Research Faculty of Medicine and Health Sciences Carl von Ossietzky University Oldenburg Oldenburg Germany

**Keywords:** depression, feedback, patient and public involvement, patient health questionnaire (PHQ‐9), patient participation, primary health care, qualitative research

## Abstract

**Background:**

Patient and public involvement (PPI) is increasingly required in mental health services research. To empower patients to actively address depression, the GET.FEEDBACK.GP study evaluates a patient‐targeted feedback intervention after depression screening using the Patient Health Questionnaire (PHQ‐9).

**Objective:**

To refine the patient‐targeted feedback from a previous study within a participatory research team (PRT) by conducting workshops to investigate patients' needs and preferences for feedback. To evaluate the process and outcome of PPI.

**Design:**

Patient and public involvement was carried out on the levels of collaboration and consultation. A PRT of patient partners and researchers planned and conducted three workshops with patients. Patients' needs were investigated using a focus group. Participants prioritized needs, discussed feedback drafts and evaluated two drafts using cognitive debriefings. Researchers of the PRT communicated the results at project level. PPI was evaluated using the Public and Patient Engagement Evaluation Tools (PPEET).

**Setting and Participants:**

A purposeful sampling of N = 12 patients with experiences of depression participated in at least one workshop.

**Results:**

Relevant content‐related needs about feedback (eg no distinction between severe and moderate symptoms), recommendations for action and patient‐relevant information were considered. Needs for comprehensible, valuing, nonstigmatizing language and design elements (eg dimensional bar) were implemented. Workshops and PRT were positively evaluated.

**Discussion and Conclusions:**

Patient and public involvement influenced the content, wording and design of the feedback. Strengths include two levels of PPI, methodical diversity and purposeful sampling. Limitations include the lack of inclusion of patients who are unaware of their depression. The evaluated PPI concept can be useful for future studies.

## 1 INTRODUCTION

In the field of research and development of patient‐relevant health‐care interventions, there is a traditional dominance of researchers' perspectives.^1^ Researchers often try to anticipate patients' needs and preferences from an outside perspective based on the literature and known evidence instead of including the target group in the development of interventions, technologies or patient information relevant to their care.^2^, ^3^, ^4^ Therefore, this paper describes the patient and public involvement (PPI) within the randomized controlled trial (RCT) Get.Feedback.GP (registered at ClinicalTrials.gov NCT03988985)^5^ during the intervention development phase as well as its evaluation from the perspectives of involved parties (patient partners, participants and researchers) regarding process, impacts and challenges of the PPI.

Patient and public involvement is of increasing importance in health services research to deliver health services that meet patients' needs. The United Kingdom can be seen as a model for advancing and implementing PPI in the research process.^6^ For example, the National Institute for Health Research (NIHR) is only funding research including PPI.^7^ Supported by the NIHR, INVOLVE^6^ and further initiatives, such as the Patient‐Centered Outcomes Research Institute (PCORI)^8^ in the United States and the Strategy for Patient‐Oriented Research (SPOR)^9^ in Canada, have been established to enhance PPI. Commonly, PPI is considered desirable to obtain findings and to focus on outcomes more relevant for the targeted patient group.^10^, ^11^, ^12^

In Germany, funders and researchers recognize the relevance of involving patients in health services research.^13^, ^14^ Currently, however, initiatives as internationally implemented do not exist, and systematic PPI in German health services research is scarcely practised.^15^, ^16^

While research is often *about, on* or *for* patients, PPI is defined as research *with* or *by* patients and members of the public.^17^, ^18^ Contributing on different levels, patients can provide their valuable knowledge and expertise in single research phases (consultation), collaborate continuously in a research team as patient partners (collaboration), or even initiate and conduct research independently (user‐controlled research).^18^ Patients' perspectives can be used by researchers to improve the quality and practical relevance of their research.^1^, ^19^ PPI can help to increase patient participation in studies by making study materials and recruitment procedures suitable for the investigated target group.^1^ In addition, PPI can help to make patient information material more relevant, readable and understandable to patients.^20^ Regarding implementation and dissemination of study results, positive findings of PPI were reported (eg by presenting research findings in a lay user‐friendly and poignant manner).^1^

Currently, there are some recommendations and suggestions about how to carry out and report PPI.^17^, ^21^, ^22^ However, it remains unclear how PPI, when indicated, should be conducted and evaluated systematically.^23^, ^24^ Several reviews criticize the poor and inconsistent reporting of PPI in studies (eg reporting on the representation of the involved sample^25^) and its lack of systematic evaluation,^1^, ^21^, ^26^, ^27^ which results in a weak evidence base and makes it difficult to understand how and under what circumstances PPI has an impact on research.^21^, ^28^ Furthermore, it is criticized that researchers tend to perform PPI in a tokenistic manner^1^, ^22^, ^28^ (eg to comply with study and funders' policies^1^), thus impairing the contribution and impacts of PPI on research. Studies show that the extent of PPI impact depends, for example, on chief investigators' support^28^ and preparedness to include PPI in their research,^17^ on researchers' understanding regarding the purpose of PPI^29^ and on the quality of the relationship between the researchers and involved patients.^17^, ^28^

Since PPI is a complex intervention, its evaluation is also complex and should include process, outcome and impact.^1^ Evaluating the benefits and cost‐effectiveness of PPI in research is crucial to achieving effective PPI and to identifying factors affecting the impact and thereby overcoming tokenism.^21^, ^23^

Within the field of mental health services, PPI has increased over time^30^ and can be advantageous.^31^ PPI showed positive impacts on recruitment success^30^ and on prioritization of clinically relevant outcomes on a systematic review.^32^ Gillard and colleagues^33^ found that, compared with conventional researchers, there were differences in how patient partners carried out interviews with patients and especially how they analysed the transcripts (eg with a stronger focus on patients' experiences and feelings). Additionally, in the development of technology‐based interventions for people with mental illnesses,^34^ there is evidence that involving potential users helps to design them to be more responsive and user‐friendly.^35^ Therefore, GET.FEEDBACK.GP was designed by involving patients using mental health services during the intervention development phase.

### 1.1 Get.Feedback.GP

Although recommended,^36^, ^37^, ^38^ there is a lack of international high‐quality studies proving the efficiency of a depression screening^39^ coupled with accurate diagnosis, appropriate treatment and follow‐up.^38^ The DEPSCREEN‐INFO RCT demonstrated that a patient‐targeted, written feedback intervention after depression screening with the Patient Health Questionnaire (PHQ‐9)^40^ had a positive effect on the severity of depression^41^ and cost‐effectiveness^42^ after six months in patients with coronary heart disease. The suspected underlying mechanism was that patient‐targeted feedback of the screening result directed at patients empowers them to actively manage depression. In addition to the written feedback of the screening result, the feedback also included graphical elements indicating patients' depression severity as well as depicting it in relation to the general population, recommendations for action, information about depression and health‐care services, and contact information for the local university psychosomatic outpatient clinic. Overall, these findings underline the potential for the dissemination of the feedback intervention.

Therefore, the generalizability of these results with respect to primary care will be tested in the multicentre RCT GET.FEEDBACK.GP. The RCT will test the effect of a patient‐targeted feedback intervention on depression severity. Full details of the trial can be found in the registered study protocol.^5^

In summary, PPI in mental health research can have an impact on research process, interventions, data analysis strategies and results. Overall, it is recommended to involve targeted patients as early as possible.^18^

## 2 AIMS OF PPI

By involving patients with experiences of depression, we investigated their needs and preferences for a feedback intervention after depression screening. The feedback should be comprehensible and acceptable and encourage patients to actively deal with their depression. The process, impact and outcome of PPI on patients and researchers should be satisfactory.

## 3 METHODS

### 3.1 Design

**Figure 1:**
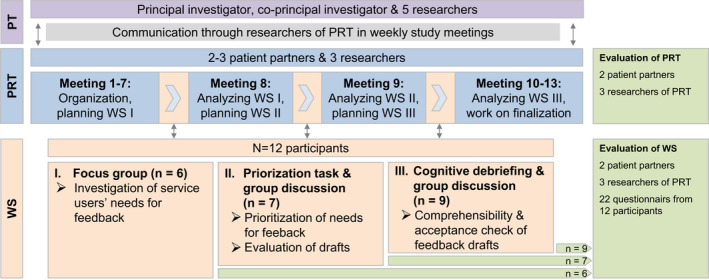
Participatory research design for the development of the feedback. Abbreviations: PRT, participatory research team; PT, project team; WS, workshop

A participatory research design involving patients with experiences of depression on the level of collaboration through the establishment of a participatory research team (PRT) and on the level of consultation using focus groups, prioritization tasks, group discussions and cognitive debriefing was chosen (Figure 1). Researchers of the PRT referred to available recommendations for PPI to prepare the PRT and the workshops.^18^

### 3.2 Participatory research team

Three patient partners with experiences of depression were recruited to the PRT to assist at all stages in the development process of the patient‐targeted feedback intervention (Figure 1). Former study participants who agreed to be contacted again for study purposes were contacted by the researchers via e‐mail and selected based on their experiences with depression and their gender. Patient partners were experienced in peer counselling (n = 1), leading support groups (n = 1) or contributing in participatory research (n = 1). The three researchers of the PRT (psychologists who work in academia for more than 10, 3 and 1 year) were experienced in PPI and patients with depressive disorders. During the planning phase, one patient partner dropped out after two months for health reasons. Patient partners continuously participated in 13 study meetings to plan the development of the feedback intervention as well as to comment on ethical factors and study course. They prepared PRT meetings (eg suggested methods and guiding questions for the workshops), with the project team (PT) deciding and specifying the workshops. In addition to planning, they were involved in conducting the three workshops and in interpreting the results. They also helped to recruit participants for the workshops. They checked comprehension of structure, content and material of the workshops from the patients' perspective. Furthermore, they contributed to the preparation of this manuscript. Patient partners received an expense allowance of 20€ per hour.

### 3.3 Workshops

The PRT conducted a series of three three‐hour workshops. Results from each workshop were included in the next workshop. Each workshop included information about the design and aims of the current study and about the RCT GET.FEEDBACK.GP. To create a trustful group atmosphere, patient partners introduced themselves and afterwards invited the participants to introduce themselves. At the end of each workshop, the PRT provided an outlook on the next workshop and asked participants to evaluate the workshop. All workshops were conducted by the PRT in the first quarter of 2019 at the Department of Medical Psychology of the University Medical Center Hamburg‐Eppendorf, Germany. The results were communicated at the project level by the researchers of the PRT in weekly trial meetings. In the **first workshop**, the PRT provided information on the project and the planned PPI. Furthermore, the PRT conducted a focus group^43^ to investigate patients' needs for feedback after the depression screening. In the **second workshop**, patients prioritized recommendations derived from the first workshop supplemented by recommendations from the literature^44^, ^45^ and from the patient partners and discussed feedback drafts. Regarding the latter, the PRT presented five feedback drafts^41^, ^46^, ^47^, ^48^, ^49^ (split into feedback on the test results and recommended actions) and asked participants to sequence them (best to worst draft). Afterwards, the two best‐rated drafts and the draft from the previous DEPSCREEN‐INFO study^41^ were discussed in the workshop. In the **third workshop**, the PRT checked the comprehensibility and acceptance of two feedback drafts (Appendix 1), using cognitive debriefings^50^ in five groups of one to two participants, each led by one member of the PRT. The two feedback drafts were first drafted by the PRT, reviewed and approved by the project team and then finalized by the PRT. In addition, the participants discussed two graphic representations of the feedback (dimensional bar figure vs traffic light figure, Appendix 1) and different designs (one‐sided feedback vs feedback folded in half with the text on the inside) in the group.

### 3.4 Participants

Participants were included if they had recent or past experiences with a depression diagnosis. The PRT aimed to include patients' differing with respect to age, gender and date of first depression diagnosis to include different experiences (purposeful sampling^51^). Furthermore, we aimed to include patients from different care settings. Researchers recruited the participants through different approaches such as a primary health‐care centre, an outpatient clinic for psychosomatic medicine and psychotherapy and an outpatient clinic for geriatric psychiatry via an information sheet. Patient partners recruited participants through peer support contacts, support groups and private contacts. All participants provided written informed consent. An expense allowance of 50 € per workshop was paid to participants. The study was conducted in accordance with the Helsinki Declaration and was approved by the Ethics Committee of the Medical Chamber of Hamburg (reference number: PV5975).

### 3.5 Data collection and analyses

To investigate patients' need for a feedback intervention, participants were asked about information and layout needs during a focus group moderated by one researcher and one patient partner with three predefined questions (*workshop 1): ‘Which information did you need when you heard of your depression for the first time?’; ‘How should a written feedback on depressive symptoms look like?’; and ‘What should it contain?’* The discussion was audiotaped and transcribed. Researchers of the PRT independently derived recommendations for the feedback from the first workshop using thematic analysis.^52^ Patient partners read the transcripts and reviewed and approved these recommendations.

In the second workshop, each participant rated the importance of the recommendations (very important, almost important and not important). Afterwards, each participant ranked five feedback drafts from the best to the worst draft. In a group discussion, the two best‐rated drafts and the draft from the previous DEPSCREEN‐INFO^41^ study were discussed with respect to helpfulness (‘*What is helpful about the draft?’*), comprehensibility (‘*What is misleading/ comprehensible in the draft?’*), encouragement (‘*What is encouraging/ frightening about the draft?’*) and matching the recommendations (‘*In what way are the recommendations realized in the draft?’*). The discussion and the consensus‐based statements of the group were protocolled by the researchers of the PRT on a flipchart, while the patient partners continued the moderation. On this basis, we jointly developed new drafts for each feedback element during the workshop using ad hoc analyses. Each individual formulation was continuously adapted by the researchers on a PowerPoint screen in parallel with the discussion until no further changes were necessary from the participants' perspective.

During the third workshop, the PRT instructed participants to concurrently think aloud^53^ while reading one of the two feedback drafts (‘*Please read the feedback and say everything aloud that goes through your mind.’*). Afterwards, the interviewer prompted questions about comprehensibility (‘*Please explain in your own words what was written in the feedback’.*), intention (‘*What would you do after receiving this feedback?’*) and acceptance (‘*In what way do you think this feedback is respectful, empathic and hopeful?’*) and requested an overall rating (school grade) and suggestions for improvement. Thoughts and answers were protocolled by the interviewer using a standardized documentation sheet including the questions mentioned above. Participants' rankings were analysed descriptively. The content of the discussions and comments in the cognitive debriefings were organized within a matrix and analysed by the PRT.

Participants evaluated format and structure, characteristics of the moderators as well as the scope and meaning of all workshops with an adapted standardized tool for teaching evaluation^54^ used in the study by Brütt et al^32^ The items were adjusted to the context of the workshops. For evaluating the key aspects of PPI, different tools to evaluate PPI in research and health system decision making^55^ can be used. For process (eg communication and support for participation) and outcome evaluation (eg impacts and influence of PPI) of the engagement components (workshops and PRT), we used a German translation of the Public Patient Engagement Evaluation Tool (PPEET).^56^ Participants filled in the participant questionnaire for one‐time engagement activities at the end of each workshop. Patient partners as well as researchers of the PRT filled in either the participant questionnaire for ongoing engagement or the project questionnaire for the evaluation of the workshops and the PRT (Figure 1). Researchers of the PRT analysed questionnaire data quantitatively regarding frequencies using PASW Statistics 18.^57^ All relevant aspects of process, outcome and impact of PPI are reported following the recommendations of the GRIPP2 reporting checklists.^21^

## 4 RESULTS

### 4.1 Participants

Twelve patients with experiences of depression participated in the workshops (first workshop, n = 6; second workshop, n = 7; third workshop, n = 9). They were between 24 and 81 years old (median 60); seven were female, and five were male; and three were born abroad. Four held an A level (‘Abitur/Fachabitur’), four had graduated from middle school (‘Realschule’), one had not graduated, and three were missings. Participants' first diagnosis of depression was one to 49 years ago (median 11). Eight participants reported currently suffering from depression, and ten reported suffering from comorbidities (n = 5, mental illness; n = 9, physical illness). Participants had sought treatment from general practitioners (GPs, n = 9) and had undergone psychotherapy (n = 10) or psychopharmacotherapy (n = 5). They were recruited through patient partners (n = 5) as well as through researchers via an outpatient clinic for psychosomatic medicine and psychotherapy (n = 1), an outpatient clinic for geriatric psychiatry (n = 4) and a primary health‐care centre (n = 2).

### 4.2 Recommendations for the feedback intervention (workshop 1 and 2)

Recommendations for the patient‐targeted feedback intervention included content‐related aspects, language and design elements.

#### 4.2.1 Content‐related recommendations

Participants mentioned content‐related recommendations for the feedback of the PHQ‐9 results and recommendations for action and ranked them according to their importance (Table 1).

**Table 1:** Prioritized content‐related recommendations concerning feedback of the results and recommended actions in the feedback intervention (n = 6)

Feedback on the PHQ‐9 results should …	n ‘very important’	n ‘almost important’	n ‘not important’
1. … foster problem perception.	7	0	0
2. … refer to own depressive symptoms.	6	1	0
3. … contain the recommendation to speak directly to the GP about possible actions.	6	1	0
4. … contain the information that the PHQ‐9‐result does not replace any diagnosis and that it is necessary to clarify the depressive symptoms.	5	1	1
5. … convey confidence and hope.	4	3	0
6. … explicitly name depression to provide patients the opportunity to deal with it.	4	2	1
7. …. not contain stigmatizing words (eg crisis, depression) to encourage patients to speak to their GP first.	2	4	1
8. … contain support in the event of difficulties in implementing actions (eg lethargy impeding help‐seeking).	2	3	2
9. … contain different preventive offers and treatment options.	2	2	3
10. … encourage patients to seek professional help (even if it is difficult) and should emphasize the GPs' competence.	1	5	1
11. … contain concrete next steps instead of a list of treatment recommendations.	1	0	6
12. … contain what patients can do for themselves (besides guideline‐based treatment recommendations).	0	5	2

Results from the cognitive debriefings showed that participants preferred feedback on ‘depressive complaints’ instead of either ‘moderate depressive symptoms’ or ‘severe depressive symptoms’ because the distinction of severity led to different interpretations and confusion. In addition, participants' responses indicated that the feedback on the PHQ‐9 should be formulated carefully (eg ‘your answers give a hint…’ instead of ‘result: you are highly likely to have…’). Furthermore, participants recommended providing patient‐relevant destigmatizing information about depression including Internet addresses with trustworthy information and addresses of local health services as well as a low threshold and 24‐h‐available telephone helpline.

#### 4.2.2 Language‐related recommendations

Participants agreed with language‐related recommendations from the literature about short sentences^58^ and simple, concrete and inclusive language (eg related to gender^45^). They emphasized the importance of a comprehensible, valuing, and nonclinical and nonstigmatizing wording focused on the patient.

#### 4.2.3 Design‐related recommendations

To avoid stigmatization, participants preferred a feedback flyer containing the information on the inner side that was not visible to other patients in the waiting room. To illustrate the results of the PHQ‐9, participants preferred the dimensional bar figure over the traffic light and prevalence of depression figure because the latter reinforces the impression of a depression diagnosis and of doing something wrong. Participants preferred a bar with smooth transitions between equal red, yellow and green parts to illustrate depressive symptoms as a continuum.

### 4.3 Comprehensibility and acceptance of two feedback drafts (workshop 3)

Participants understood the message of both drafts (Appendix 1). While they had no preference regarding both drafts on severe depressive symptoms (average grades 3.5; from 1=‘very good’ to 6=‘deficient’), they preferred draft B (average grade 2) compared with draft A (average grade 4.5) on moderate depressive symptoms. Four (draft B) or three (draft A) out of five participants would speak to the GP about the feedback. Participants stated that draft B is formulated as ‘hopeful because it shows the option of treatment’ and conveyed a ‘feeling of being taken seriously’. However, they would prefer personal feedback from their GPs.

### 4.4 Evaluation of PPI

#### 4.4.1 Impacts

**Figure 2:**
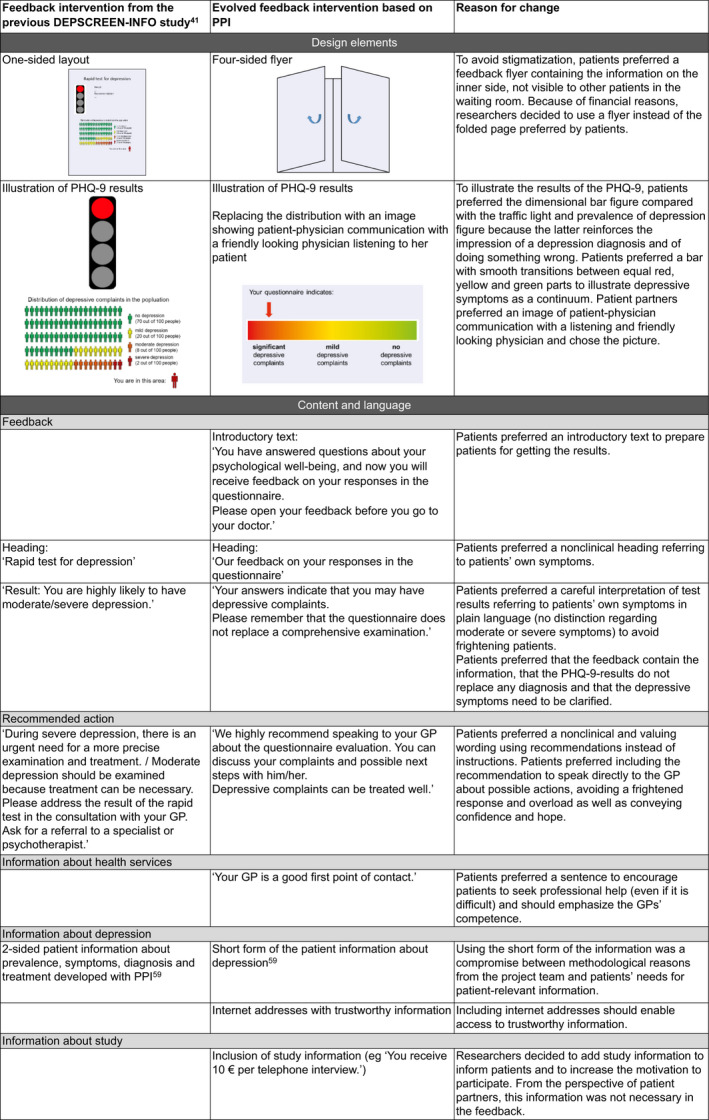
Impacts of patient and public involvement on the feedback intervention

Figure 2 shows the impacts of PPI on the development of the feedback intervention.

Nevertheless, some needs (eg preference for personal feedback, inclusion of telephone helpline) could not be realized because of the study design or lack of evidence. The final feedback is available from the authors upon request.

#### 4.4.2 Workshop evaluation

Participants rated the workshops' format and structure, characteristics of the moderators as well as the scope and meaning of all three workshops on a five‐point Likert‐scale from ‘strongly disagree’ to ‘strongly agree’ (Appendix 2). They agreed and strongly agreed with the workshops’ clear structures (item 2), moderators' openness to criticism (item 5), friendliness (item 7) and helpfulness (item 8). They moderately to strongly agreed with the illustrations used in the workshops (item 1), moderators fostering debates about the topics (item 6) and the meaning of the topics (item 9). One participant in workshops 2 and 3 disagreed that the workshops had an adequate pace (item 10), and approximately half of the participants rated the number of topics covered in the workshops as too many (item 11).

**Figure 3:**
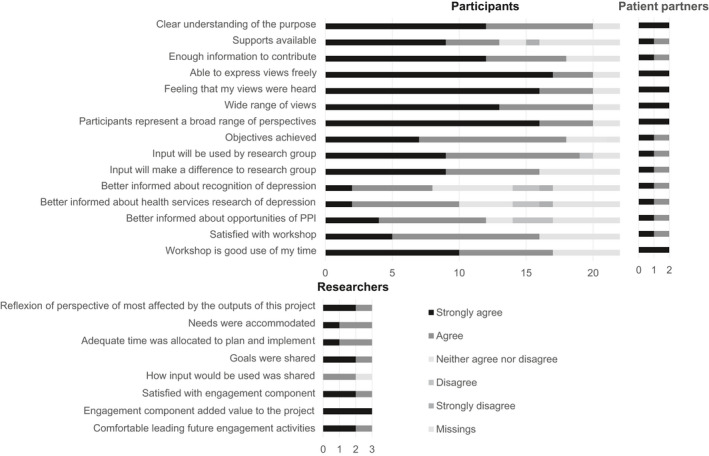
Summarized evaluation (PPEET) of all three workshops from the perspective of participants (data from 22 questionnaires [workshop 1: n = 6; workshop 2: n = 7; workshop 3: n = 9]), patient partners (n = 2) and researchers from the PRT (n = 3)

Regarding process and outcome evaluation, participants as well as patient partners rated the communication and support for participation as well as impacts and influence of the workshops, whereas researchers of the PRT rated the integrity of the design and process (PPEET; Figure 3, Appendices 3, 4, 5).

Participants appreciated the exchange, the respectful conversation and the moderation through patient partners (eg felt more accepted, good atmosphere). However, participants saw needs for improvement regarding possibilities to prepare themselves, moderation and organizational aspects (eg regional catering). Patient partners emphasized that they were integrated in the workshop group and that they had enough space to represent their own position. From their point of view, workshops were helpful for the development of the feedback and characterized by creativity and integration of patients' different perspectives.

#### 4.4.3 Participatory research team evaluation

Patient partners and researchers of the PRT were satisfied with the team and thought that it was valuable for the project (Appendices 4 and 5). From the patient partners' perspective, the communication and support for participation as well as impacts and influence of the workshops regarding the feedback were positive (Appendix 4). Researchers rated the integrity of the design and process of the PRT mainly positively (Appendix 5). Patient partners reported their initial experience of being confused because of ‘several unknown terms’. They rated teamwork as ‘on an equal footing’ and emphasized the strength of the team to take into account different aspects and perspectives. They identified a need of a written contract and of more time and structure for the preparation of the workshops. Researchers were interested in developing PPI‐related skills regarding PPI during proposal writing, realistic expectations of patients' resources and competencies, and guidelines and templates for organizational aspects of PPI (contracts, payment, data protection, authorships, acknowledgement, rights and obligations).

## 5 DISCUSSION

Patient and public involvement carried out at the levels of collaboration (PRT) and consultation (workshops) led to changes regarding content, language and design of the patient‐targeted feedback intervention. For instance, content‐related needs of patients included the fostering of problem perception, reference to their own symptoms, careful interpretation of test results, recommendation to speak directly to the GP and conveyance of confidence and hope. Patients preferred plain, valuing and nonstigmatizing language. Furthermore, they favoured figures that illustrate symptoms as a continuum as well as a design, which enables them to read the feedback privately. These needs were considered in the development of the feedback intervention. Only some of the patients' preferences (eg recommendation of telephone service; personal instead of written feedback) could not be realized because of clinical reasons and aspects of the study design. We regard the development of the feedback intervention as participatory because we implemented most of the recommendations of the target group that did not conflict with known evidence. Overall, the participants as well as patient partners and researchers from the PRT were satisfied with the structure, the opportunities to get involved and impacts of PPI on the research project.

Our results align with the findings of previous studies in the field of technology‐based mental health interventions, namely, that patients prefer short sentences,^58^ visual components^60^ and inclusive (eg relating to gender^45^) and nonclinical language to avoid stigma.^44^, ^61^ For instance, the participants in our study preferred a loss of information about severity of depressive symptoms (‘moderate’ or ‘severe’) over the usage of clinical language. By including a section with information about depression on the feedback flyer, we met patients' needs regarding psychoeducation^35^, ^61^ and the provision of more in‐depth information, which is also recommended in the literature.^60^

The implementation of the results of PPI at the project level has been subject to several factors. Requirements of the RCT, evidence base and financial aspects restricted the implementation of patients' recommendations. These restrictions should be clarified at the beginning to define the scope of PPI. Moreover, delays, time pressure due to formal requirements (eg ethical approval) as well as project deadlines had an impact on the process and implementation of PPI.

According to the evaluation results, the researchers were perceived as respectful and supportive of PPI. It is known that the impact of PPI can be increased if principal investigators and researchers have a supportive attitude towards PPI methods and their use in accordance with the intended purpose.^17^, ^28^, ^29^ Conventionally, academic researchers and health professionals are accustomed to having control over what and how research should be conducted, and the involvement of patients will necessarily change this balance.^1^ However, PPIs' potential to generate valuable contributions to research should be given more weight than the hurdle of sharing decision power. The evaluation results may indicate that research training (eg regarding scientific wording, planning and analysis methods) for patient partners and researchers to support organizational and content‐related aspects of PPI can be useful. There is some evaluated research training for patients with mental disorders^62^, ^63^; however, to our knowledge, there is a lack of training for researchers on PPI. As Høeg et al^64^ argue, the dynamic interaction of knowledge of involved parties (researchers and patients) during a PPI activity also involves the danger of interpersonal tensions. This can also be addressed through collaboratively developed specific guidelines and training.^29^, ^64^

### 5.1 Strengths and limitations

We have reported all relevant aspects of process, outcome and impact of PPI following the recommendations of the GRIPP2 reporting checklists.^21^ Our PPI process was in accordance with the recommendations of the INVOLVE initiative for implementation.^18^ Our PPI study met the following six conditions identified by the RAPPORT study,^65^ which influence the positive outcomes and impacts of PPI. Involved service patients had a strong attachment to the targeted study population (1), the research team had a positive attitude towards PPI input (2), both researchers and patients invested in a good relationship (3), and the PPI was evaluated through a systematic approach (4). Instead of only one recommended key person (5),^65^ all three researchers of the PRT coordinated PPI in this study and communicated the PPI results. This enabled a PPI of high quality, without major changes within the course of the usual project work. In accordance with the RAPPORT study, researchers and the involved patients generally had a shared understanding of the moral and methodological purposes of PPI (6); minor differences cannot be avoided.

One strength of this study is that we involved patients on the levels of consultation and collaboration during the feedback development. Regarding PPI in research, various constellations of patient partners and researchers, participation levels as well as stages of involvement can be found in the literature.^21^, ^28^, ^32^ The evaluation results indicated that our group constellation within the PRT, in which researchers and patients were equally represented, and the comoderation of the workshops through the patient partners created a supportive environment in which patients felt encouraged to express their opinions and share their ideas. As recommended,^18^ patients were involved in the early stages of the project, which allowed them to not only deliver valuable information for the development of the feedback intervention but also to collaborate in planning the workshops (eg sampling strategy, workshop content).

Our sample size of the involved participants is comparable to similar studies that considered users' perspectives during the development of technology‐based interventions (eg smartphone application) for mental and other illnesses.^34^, ^66^, ^67^ Furthermore, the methodical diversity within the workshops should be highlighted. We were able to apply different qualitative methods efficiently (eg focus group and cognitive debriefings with an appropriate group size). Through purposeful sampling, views from patients with different experiences of depression and health services, different ages and genders were taken into account. The dropout of one patient partner emphasizes the importance of patients' needs and expectations of involvement.^22^, ^28^ Although the understanding of roles, expectations and working effort were clarified at the first PRT meetings, we recommend better alignment of expectations and requirements and evaluation of them steadily.

By using the PPEET, a multiperspective evaluation of the different PPI components could be realized, and benefits and challenges were identified. The former experience in PPI of two researchers of the PRT likely facilitated the development of positive relationships and working methods, making the PPI feasible and of high quality.

However, our study has some limitations. For methodical and ethical reasons, it was not possible to include patients not knowing about their own depression. As a consequence, the involved patients did not represent the entire future target group for the feedback intervention.^68^ When checking the comprehensibility and acceptance of the two feedbacks, five out of nine participants took part in at least one of the previous workshops. Because they already commented on content‐, language‐ and design‐related aspects of the feedback, their initial reaction may have been biased. In addition, no formal training for researchers and patient partners was conducted at the trial outset. Finally, it should be mentioned that we started PPI after the funding for the multicentre RCT GET.FEEDBACK.GP has been granted. The scope of PPI was determined as the development of a feedback intervention based on the feedback intervention from the DEPSCREEN‐INFO study.^41^ PPI at an earlier stage (eg when writing the funding application) might have opened up more possibilities to develop a new intervention from scratch or even the RCT design.

Even though PPI is demanded by some funders,^13^, ^69^ there is a lack of essential strategic and infrastructural support of PPI^10^ in German in mental health research.^62^ While consultative patient participation is practised sporadically, we have no knowledge of involvement initiatives in German mental health research that systematically apply the concept of collaborative involvement of patients in the research process.^15^ Further changes in the funding structures are necessary, which should meet the requirements (eg establish participatory advisory group) and facilitating factors of participatory research.

### 5.2 Implications

It is known that PPI can increase the practical relevance of outcomes,^1^ ecological validity,^19^ patient participation in studies by making study materials understandable^1^, ^20^ and recruitment procedures suitable^1^ for the investigated target group. PPI enables direct integration of patients' needs into interventions. Nevertheless, further evidence‐based studies are needed to assess whether PPI improves the effectiveness of interventions. There is a crucial difference between making research more lay‐friendly or patient‐targeted and collaborating with patients on an equal footing and integrating their perspectives. For each project, it must be carefully considered how the results of PPI can be implemented. Evaluated PPI concepts are essential for participatory health‐care research. Our structured and evaluated PPI concept (eg different levels of participation, coordination and communication through researchers) can be used for the implementation of PPI within intervention development for addressing mental disorders. Although we systematically evaluated PPI using the PPEET, significant aspects that are more noticeable by using narrative approaches have not been investigated. Based on our reflection on the process, we identified the benefits of letting independent researchers coordinate the PPI process, and we recommend learning from each other and carefully reflecting on the PPI process to avoid tokenistic forms of research involvement. Additionally, we suggest that research training should be offered to involved patients to maximize their contribution and impact on research, as well as training for researchers for developing PPI‐related skills.

The effectiveness of the participatory‐developed feedback intervention after depression screening will be tested by the multicentre RCT GET.FEEDBACK.GP. The data collection started in July 2019 and is expected to be completed in July 2021. Patient partners and participants will be involved at the end of the RCT to create a report of the results that is comprehensible to patients.

## CONFLICT OF INTEREST

There are no conflicts of interest.

## Data Availability

The data that support the findings of this study are available from the corresponding author upon reasonable request. The data are not publicly available due to ethical or privacy restrictions.

## Appendix 1

### Feedback drafts developed based on workshops 1 and 2, evaluated in workshop 3

*Note*: Drafts for the feedback of moderate severe depressive symptoms (PHQ‐9: 10 to 14 points) and adjustment for the feedback of severe depressive symptoms (PHQ‐9: 15‐27) marked by squared brackets. The traffic light and the dimensional bar figures illustrate the feedback of severe depressive symptoms.

## Appendix 2

### Workshop evaluation from the perspective of workshop participants (frequencies, 1. workshop N = 6; 2. workshop N = 7; 3. workshop; N = 9; English translation from Brütt et al., 2017; adapted from Zumbach et al., 2007)

**Table:**

Item	Workshop	Strongly disagree	Disagree	Almost agree	Agree	Strongly agree	Missings
*Format and structure*							
1. The subject was illustrated adequately (eg through examples, visualizations).	1	—	—	—	3	3	—
	2	—	—	1	4	1	1
	3	—	—	1	3	5	—
2. The workshop was clearly structured.	1	—	—	—	5	1	—
	2	—	—	—	5	1	1
	3	—	—	1	2	6	—
3. The learning goals of the workshop were clearly defined.	1	—	—	—	1	5	—
	2	—	—	—	2	3	2
	3	—	1	1	1	5	1
4. Additional helpful resources were given (eg, handset, literature, internet access etc).	1	2	1	—	1	—	2
	2	3	—	1	—	—	3
	3	1	2	1	4	—	1
*Characteristics of the lecturers*							
5. The lecturers were open to criticism.	1	—	—	—	2	3	1
	2	—	—	—	2	4	1
	3	—	—	—	3	6	—
6. The lecturers encouraged to critically deal with the topics covered.	1	—	—	—	3	3	—
	2	—	—	2	3	1	1
	3	—	1	—	1	7	—
7. The lecturers were friendly and open in dealing with the group.	1	—	—	—	—	6	—
	2	—	—	—	—	6	1
	3	—	—	—	1	8	—
8. The lecturers were helpful.	1	—	—	—	1	5	—
	2	—	—	—	1	5	1
	3	—	—	—	1	8	—
*Scope and meaning*							
9. The meaning of the topics covered was high (eg, for peer counseling etc).	1	—	—	—	3	2	1
	2	—	—	2	3	1	1
	3	—	—	—	3	3	3
10. The content of the workshop was covered in an adequate pace.	1	—	—	—	—	6	—
	2	—	1	—	4	1	1
	3	—	1	—	1	7	—
11. The amount of topics covered in this workshop was too comprehensive.	1	1	2	—	—	3	—
	2	1	1	3	1	—	1
	3	1	4	1	1	2	—

## Appendix 3

### Workshop evaluation from the perspective of participants and patient partners (frequencies, 1. workshop N = 6; 2. workshop N = 7; 3. workshop; N = 9; patient partners N = 2, participant questionnaire: module A and B of the PPEET from Abelson et al., 2016)

**Table:**

Item	Work‐ shop	Participants (WP) patient partners (PP)	Strongly disagree	Disagree	Neither agree nor disagree	Agree	Strongly agree	Missings
*Communication and supports for participation*								
1. I had a clear understanding of the purpose of the workshops.	1	WP	—	—	—	3	3	—
	2	WP	—	—	—	3	3	1
	3	WP	—	—	—	2	6	1
	—	PP	—	—	—	—	2	—
2. The supports I needed to participate were available (eg, travel, childcare).	1	WP	—	—	—	2	4	—
	2	WP	1	—	1	1	2	2
	3	WP	—	—	1	1	3	4
	—	PP	—	—	—	1	1	—
3. I had enough information to contribute to the topic being discussed.	1	WP	—	—	1	1	4	—
	2	WP	—	—	1	3	2	1
	3	WP	—	—	—	2	6	1
I have enough information to be able to carry out my role.	—	PP	—	—	—	1	1	—
*Sharing your views and perspectives*								
4. I was able to express my views freely.	1	WP	—	—	—	—	6	—
	2	WP	—	—	—	3	3	1
	3	WP	—	—	—	—	8	1
	—	PP	—	—	—	—	2	—
5. I feel that my views were heard.	1	WP	—	—	—	—	6	—
	2	WP	—	—	—	2	4	1
	3	WP	—	—	—	2	6	1
	—	PP	—	—	—	—	2	—
6. A wide range of views on the topics discussed was shared.	1	WP	—	—	—	2	4	—
	2	WP	—	—	—	2	4	1
	3	WP	—	—	—	3	5	1
	—	PP	—	—	—	—	2	—
7. The individuals participating in the workshops represent a broad range of perspectives.	1	WP	—	—	—	1	5	—
	2	WP	—	—	—	2	4	1
	3	WP	—	—	—	1	7	1
	—	PP	—	—	—	—	2	—
*Impacts and influence of the workshops*								
8. I think that the workshops achieved their objectives.	1	WP	—	—	1	3	2	—
	2	WP	—	—	1	4	1	1
	3	WP	—	—	1	4	4	—
The workshops are achieving their stated objectives.	—	PP	—	—	—	1	1	—
9. I am confident the input provided through the workshops will be used by the research group.	1	WP	—	1	—	2	3	—
	2	WP	—	—	—	3	3	1
	3	WP	—	—	—	5	3	1
I am confident that the project group takes the feedback provided by the workshops into consideration.	—	PP	—	—	—	1	1	—
10. I think the input provided through the workshops will make a difference to the work of the research group.	1	WP	—	—	—	2	3	1
	2	WP	—	—	—	3	1	3
	3	WP	—	—	—	2	5	2
I think that the work of the workshops makes a difference to the work of the research group.	—	PP	—	—	—	1	1	—
*Final thoughts*								
11. As a result of my participation in the workshops, I am better informed about the recognition of depression.	1	WP	—	1	1	2	1	1
	2	WP	—	1	2	1	—	3
	3	WP	1	—	3	3	1	1
	—	PP	—	—	—	1	1	—
12. As a result of my participation in the workshops, I am better informed about health services research of depression.	1	WP	—	1	1	2	1	1
	2	WP	—	1	2	1	—	3
	3	WP	1	—	1	5	1	1
	—	PP	—	—	—	1	1	—
13. As a result of my participation in the workshops, I am better informed about opportunities of patient and public involvement.	1	WP	—	1	—	3	1	1
	2	WP	—	1	1	2	—	3
	3	WP	—	1	1	3	3	1
	—	PP	—	—	—	1	1	—
14. Overall, I was satisfied with the workshops.	1	WP	—	—	—	3	2	1
	2	WP	—	—	—	3	1	3
	3	WP	—	—	—	5	2	2
	—	PP	—	—	—	1	1	—
15. The workshops were a good use of my time.	1	WP	—	—	—	1	4	1
	2	WP	—	—	—	3	1	3
	3	WP	—	—	—	3	5	1
	—	PP	—	—	—	—	2	—

## Appendix 4

### Participatory research team evaluation from the perspective of patient partners (frequencies, patient partners N = 2, participant questionnaire: module B of the PPEET from Abelson et al., 2016)

**Table:**

Item	Strongly disagree	Disagree	Neither agree nor disagree	Agree	Strongly agree	Missings
*Communication and supports for participation*						
1. I had a clear understanding of the purpose of the participatory research team.	—	—	—	—	2	—
2. The supports I needed to participate were available (eg, travel, childcare).	—	—	—	1	1	—
3. I had enough information to contribute to the topic being discussed.	—	—	—	1	1	—
4. I was able to express my views freely.	—	—	—	—	2	—
5. I feel that my views were heard.	—	—	—	—	2	—
6. A wide range of views on the topics discussed was shared.	—	—	—	—	2	—
7. The individuals participating in the participatory research team represent a broad range of perspectives.	—	—	—	—	2	—
*Impacts and influence of the participatory research team*						
8. I think that the participatory research team achieved their objectives.	—	—	—	1	1	—
9. I am confident the input provided through the participatory research team will be used by the research group.	—	—	—	1	1	—
10. I think the input provided through the participatory research team will make a difference to the work of the research group.	—	—	—	1	1	—
*Final thoughts*						
11. As a result of my participation in the participatory research team, I am better informed about the recognition of depression.	—	—	—	1	1	—
12. As a result of my participation in the participatory research team, I am better informed about health services research of depression.	—	—	—	1	1	—
13. As a result of my participation in the participatory research team, I am better informed about opportunities of patient and public involvement.	—	—	—	1	1	—
14. Overall, I am satisfied with the participatory research team.	—	—	—	1	1	—
15. The participatory research team were a good use of my time.	—	—	—	—	2	—

## Appendix 5

### Evaluation of workshops and participatory research team from the perspective of researchers (frequencies, researcher N = 3, project questionnaire: module B of the PPEET from Abelson et al., 2016)

**Table:**

Item	Workshops (WS); Participatory Research Team (PRT)	Strongly disagree	Disagree	Neither agree nor disagree	Agree	Strongly agree	Missings
*Integrity of design and process*							
1. The perspectives of those who will be most affected by the outputs of this project were reflected through those who participated in the engagement.	WS	—	—	—	1	2	—
	PRT	—	—	—	3	—	—
2. The financial, logistical and information needs of participants (eg, travel, dietary, interpretive, childcare, etc) were accommodated.	WS	—	—	—	2	1	—
	PRT	—	—	—	2	1	—
3. Adequate time was allocated to plan and implement the engagement component (workshop; participatory research team).	WS	—	—	—	2	1	—
	PRT	—	—	—	2	1	—
4. The goals for the engagement component (workshop; participatory research team) were shared with participants.	WS	—	—	—	1	2	—
	PRT	—	—	—	2	1	—
5. Participants were told how the input from the engagement component (workshop; participatory research team) would be used by the research group.	WS	—	—	—	2	—	1
	PRT	—	1	—	—	2	—
*Final reflections*							
6. Overall, I was satisfied with the engagement component of this project.	WS	—	—	—	1	2	—
	PRT	—	—	—	2	1	—
7. The engagement component (workshop; participatory research team) added value to the project it supported.	WS	—	—	—	—	3	—
	PRT	—	—	—	—	3	—
8. As a result of my involvement in the engagement component (workshop; participatory research team) associated with this project, I will be comfortable leading future engagement activities.	WS	—	—	—	1	2	—
	PRT	—	—	—	1	2	—
